# Lipidic cubic phase serial femtosecond crystallography structure of a photosynthetic reaction centre

**DOI:** 10.1107/S2059798322004144

**Published:** 2022-05-25

**Authors:** Petra Båth, Analia Banacore, Per Börjesson, Robert Bosman, Cecilia Wickstrand, Cecilia Safari, Robert Dods, Swagatha Ghosh, Peter Dahl, Giorgia Ortolani, Tinna Björg Ulfarsdottir, Greger Hammarin, María-José García Bonete, Adams Vallejos, Lucija Ostojić, Petra Edlund, Johanna-Barbara Linse, Rebecka Andersson, Eriko Nango, Shigeki Owada, Rie Tanaka, Kensuke Tono, Yasumasa Joti, Osamu Nureki, Fangjia Luo, Daniel James, Karol Nass, Philip J. M. Johnson, Gregor Knopp, Dmitry Ozerov, Claudio Cirelli, Christopher Milne, So Iwata, Gisela Brändén, Richard Neutze

**Affiliations:** a Department of Chemistry and Molecular Biology, University of Gothenburg, Lundbergslaboratoriet Box 462, 405 30 Göteborg, Sweden; b RIKEN SPring-8 Center, 1-1-1 Kouto, Sayo-cho, Sayo-gun, Hyogo 679-5148, Japan; c Institute of Multidisciplinary Research for Advanced Materials, Tohoku University, 2-1-1 Katahira, Aoba-ku, Sendai 980-8577, Japan; d Japan Synchrotron Radiation Research Institute, 1-1-1 Kouto, Sayo-cho, Sayo-gun, Hyogo 679-5198, Japan; e Department of Biological Sciences, Graduate School of Science, University of Tokyo, 2-11-16 Yayoi, Bunkyo-ku, Tokyo 113-0032, Japan; f Department of Cell Biology, Graduate School of Medicine, Kyoto University, Yoshida Konoe-cho, Sakyo-ku, Kyoto 606-8501, Japan; g SwissFEL, Paul Scherrer Institute, Forschungsstrasse 111, CH-5232 Villigen PSI, Switzerland

**Keywords:** membrane-protein crystallization, photosynthetic reaction centre, *Blastochloris viridis*, serial crystallography, lipidic cubic phase, microcrystals

## Abstract

Serial crystallography is a rapidly growing method that can yield structural insights from microcrystals that were previously considered to be too small to be useful in conventional X-ray crystallography. Here, conditions for growing microcrystals of the photosynthetic reaction centre of *Blastochloris viridis* within a lipidic cubic phase (LCP) crystallization matrix that employ a seeding protocol utilizing detergent-grown crystals with a different crystal packing are described. LCP microcrystals diffracted to 2.25 Å resolution when exposed to XFEL radiation, which is an improvement of 0.15 Å over previous microcrystal forms. Ubiquinone was incorporated into the LCP crystallization media and the resulting electron density within the mobile Q_B_ pocket is comparable to that of other cofactors within the structure. As such, LCP microcrystallization conditions will facilitate time-resolved diffraction studies of electron-transfer reactions to the mobile quinone, potentially allowing the observation of structural changes associated with the two electron-transfer reactions leading to complete reduction of the ubiquinone ligand.

## Abstract 2

Serial crystallography relies upon the growth of microcrystals at high concentration. An LCP crystallization protocol for the *Blastochloris viridis* photosynthetic reaction centre that utilizes seeding from detergent-grown crystals is reported.

## 1. Introduction

Life on Earth depends upon the energy harvested from sunlight by photosynthesis, which incorporates a set of chemical reactions that combine to generate a versatile form of chemical energy for the cell. The core light-driven electron-transfer reactions are shared by all plants, algae, cyanobacteria and photosynthetic bacteria (Nowicka & Kruk, 2016 ▸; Barber, 2017 ▸). In plants, algae and cyanobacteria, photosynthesis is based on the absorption of photons by two photosystems (Photosystems I and II), which act in sequence to transfer electrons from water to ferredoxin. These light-driven electron-transfer reactions build up a proton concentration gradient across the energy-transducing chloroplast membrane, which is utilized in the production of ATP or is coupled to other cellular transport processes. Since the photo-oxidized special pair of tightly interacting chlorophylls of Photosystem II are reduced by the oxygen-evolving complex, which is in turn reduced by electrons extracted from water, molecular oxygen is released into the atmosphere as a side-product of this process.

In purple photosynthetic bacteria, photons are absorbed by the photosynthetic reaction centre (RC), which is closely related to Photosystem II but lacks an oxygen-evolving complex (Martin *et al.*, 2018 ▸). The RC from *Blastochloris viridis* (RC_*vir*_) was the first membrane-protein structure to be solved by X-ray crystallography (Deisenhofer *et al.*, 1984 ▸, 1985 ▸) and has been extensively used as a model protein for studying the light-driven charge-transfer reactions of photosynthesis. Subunits L and M of RC_*vir*_ each contain five transmembrane helices that form a heterodimer supporting four bacteriochlorophyll cofactors, two pheophytins, a nonhaem iron and a tightly bound menaquinone arranged about an axis of twofold pseudosymmetry. The transmembrane domain of RC*_vir_* is capped on the periplasmic side by the single transmembrane helix-containing subunit H and is capped on the cytoplasmic side by the cytochrome subunit C, which contains four haem cofactors. A photon absorbed at a special pair of bacteriochlorophylls with an absorption maximum of 960 nm (P_960_) causes this group to become photo-oxidized. The released electron moves along the A-branch (supported by the L subunit) of the cofactors *via* a bacteriochlorophyll (BChl_L_) and a bacteriopheophytin (BPhe_L_), before reaching the tightly bound menaquinone (Q_A_) and finally transferring to the mobile ubiquinone (Q_B_) of the M-branch, which is the final electron acceptor. 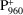 is in turn reduced from the C subunit and a second photon-absorption event triggers a second electron transfer to Q_B_. After protonation from the periplasm, the fully reduced ubiquinone is released into the membrane as ubiquinol (QH_2_). Electrons are cycled through a cytochrome *bc*_1_ complex and a soluble cytochrome *c*_2_ before returning to reduce the C subunit of RC*_vir_*. This process creates a proton gradient across the membrane as two protons are effectively moved from the cytoplasmic to the periplasmic side for every absorbed photon (Leonova *et al.*, 2011 ▸; Hunter *et al.*, 2009 ▸).

Serial crystallography is a rapidly growing field of protein crystallography that was initially developed for X-ray free-electron lasers (XFELs; Chapman *et al.*, 2011 ▸; Schlichting, 2015 ▸) but is increasingly being used at synchrotron-radiation facilities (Pearson & Mehrabi, 2020 ▸). Diffraction images are collected from a sequence of microcrystals, all with random orientations, and all diffraction images are subsequently merged together in order to recover a complete diffraction data set. A key advantage of this method is that diffraction data can be collected at room temperature and with minimal radiation damage since the same microcrystal is usually sampled only once. When a pulsed laser is used to activate a light-sensitive sample, protein structural movements can be followed with time. Light-induced structural changes in several proteins have been studied in this way at XFELs on timescales from sub-picosecond to millisecond (Tenboer *et al.*, 2014 ▸; Barends *et al.*, 2015 ▸; Pande *et al.*, 2016 ▸; Nango *et al.*, 2016 ▸; Suga *et al.*, 2017 ▸, 2019 ▸; Coquelle *et al.*, 2018 ▸; Nogly *et al.*, 2018 ▸; Kern *et al.*, 2018 ▸; Branden & Neutze, 2021 ▸; Poddar *et al.*, 2022 ▸)

Several challenges emerge when working with microcrystals relative to more conventional single-crystal cryo-crystallo­graphy. Since microcrystals are very small, which is usually an advantage when initiating a reaction within microcrystals for time-resolved diffraction studies (Branden & Neutze, 2021 ▸), their diffraction power may suffer and this is only partially overcome by using high-brilliance XFEL sources. Although larger microcrystals frequently diffract better than smaller microcrystals and may be preferable for data collection at synchrotron-radiation sources, the microcrystal density is necessarily lower for larger crystals and larger crystals are more likely to block the flow of sample injection. Various sample-injector systems have been developed for serial crystallography studies. The most popular systems used in time-resolved serial femtosecond crystallography (TR-SFX) or time-resolved serial synchrotron crystallography (TR-SSX; Pearson & Mehrabi, 2020 ▸) studies are the gas dynamical virtual nozzle (GDVN) for liquid samples (DePonte *et al.*, 2008 ▸; Tono, 2017 ▸; Weierstall *et al.*, 2012 ▸) and a viscous injector for semi-solid samples consisting of crystals in lipidic cubic phase (LCP) or grease (Weierstall *et al.*, 2014 ▸; Sugahara *et al.*, 2015 ▸; Shimazu *et al.*, 2019 ▸.) Both systems have their advantages and drawbacks. Liquid microjet slurries can be concentrated, which improves the hit rate, but the high flow rate of liquid injectors often means that it is necessary to prepare several grams of protein for a single time-resolved diffraction experiment. For very high repetition-rate XFEL studies, the high speed of the liquid injector is essential (Gisriel *et al.*, 2019 ▸; Wiedorn *et al.*, 2018 ▸; Grünbein *et al.*, 2018 ▸). Conversely, one compelling advantage of viscous microjet injectors (Weierstall *et al.*, 2014 ▸; Sugahara *et al.*, 2015 ▸) is that the extruded sample moves much more slowly than when using a GDVN injector and therefore the rate of sample consumption can be orders of magnitude lower than when using a liquid-injection system. This factor is particularly important for serial crystallography studies at synchrotron-radiation sources, since a GDVN system injects samples too rapidly across an X-ray beam for normal synchrotron-radiation monochromatic data-collection applications and therefore a viscous injector is preferred (Nogly *et al.*, 2015 ▸; Pearson & Mehrabi, 2020 ▸).

We have previously reported two liquid microcrystalline forms of RC*_vir_*, both of which have been successfully injected across an XFEL beam using a GDVN (Johansson *et al.*, 2012 ▸, 2013 ▸; Dods *et al.*, 2017 ▸). We first utilized lipidic sponge phase (LSP) microcrystals of RC*_vir_*, which provides a swollen liquid analogue of the LCP crystallization matrix but with larger aqueous channels of the mesophase; this gives it a liquid consistency (Wadsten *et al.*, 2006 ▸). This crystal form suffered from a low hit rate and our SFX LSP structure of RC*_vir_* was only determined to 3.5 Å resolution.(Johansson *et al.*, 2012 ▸, 2013 ▸) We then developed microcrystals using classical detergent-solubilized protein and crystals were grown using vapour diffusion. Iterative rounds of seeding improved the diffraction quality of these microcrystals to 2.4 Å resolution (Dods *et al.*, 2017 ▸). This crystal form was also used in TR-SFX studies of light-induced structural changes in RC*_vir_* in the picosecond time domain (Dods *et al.*, 2021 ▸). From these data, movements of the tightly bound menaquinone (Q_A_) were observed 300 ps after photo-activation of RC*_vir_*, and the protein was observed to adjust its structure to stabilize the charge-separated state. These TR-SFX studies built on earlier time-resolved Laue diffraction studies of RC*_vir_*, which revealed that Tyr162_L_ moves closer to 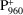 3 ms after photoactivation (Wöhri *et al.*, 2010 ▸).

Here, we developed an LCP microcrystal form of RC*_vir_* with the goal of exploring TR-SSX studies on slower timescales using synchrotron-radiation sources (Pearson & Mehrabi, 2020 ▸). LCP crystallization was initially developed for structural studies of bacteriorhodopsin (Landau & Rosenbusch, 1996 ▸) and has become one of the most successful methods when crystallizing integral membrane proteins. SFX studies of LCP-grown membrane-protein crystals have been a successful strategy for bacteriorhodopsin (Nango *et al.*, 2016 ▸; Nogly *et al.*, 2015 ▸, 2018 ▸) and G-protein coupled receptors (Weierstall *et al.*, 2014 ▸; Liu *et al.*, 2013 ▸). Since RC*_vir_* loses the mobile ubiquinone (Q_B_) during purification (Gast *et al.*, 1985 ▸), we reasoned that another crystal form which supported the addition of ubiquinone may allow two-laser-flash time-resolved diffraction studies of the reduction and protonation of ubiquinone in microcrystals of RC*_vir_*. We therefore incorporate ubiquinone 1 (UQ1) and ubiquinone 2 (UQ2) into the crystallization matrix to replace the native ubiquinone 9 that is lost during purification. One highly unusual aspect of our approach is that we utilize microcrystals resulting from a vapour-diffusion crystallization protocol (Dods *et al.*, 2017 ▸), which have a different crystal packing, as a seed stock for LCP crystallization. This step was important to obtain a reliable LCP crystallization protocol, and microcrystals were thus grown and delivered into an XFEL beam at both SACLA and SwissFEL, yielding SFX structures at 2.4 and 2.25 Å resolution, respectively. The electron density observed within the Q_B_ pocket was of similar quality to that of other cofactors, establishing that the addition of lipid-soluble cofactors is a viable strategy in LCP crystallization. Our LCP microcrystals therefore provide an alternative strategy for future TR-SFX and TR-SSX studies of the structural response of the protein to photo-activation, potentially addressing concerns about the photo-excitation conditions (Miller *et al.*, 2020 ▸; Branden & Neutze, 2021 ▸) used in earlier time-resolved diffraction studies (Dods *et al.*, 2021 ▸; Wöhri *et al.*, 2010 ▸).

## 2. Methods

Photosynthetic reaction centres from *B. viridis* were purified as described previously (Dods *et al.*, 2017 ▸; Wöhri *et al.*, 2009 ▸). *B. viridis* cells were grown anaerobically in the dark for 36 h followed by illumination for 48 h to induce protein expression. These cells were then harvested in a JLA-8.1000 rotor at 7000 rev min^−1^ for 15 min and ∼20 g of cells were broken by three sonication cycles followed by centrifugation in a JA-25.50 rotor at 15 000 rev min^−1^ for 20 min to recover the membranes in each round. Membranes were collected by ultracentrifugation in a Ti45 rotor at 45 000 rev min^−1^ for 75 min and resuspended in 20 m*M* Tris–HCl pH 8.5 followed by stirring overnight at 4°C. The OD_1012_ of the membranes was adjusted to 100 and the protein was solubilized by the addition of lauryldimethylamine-*N*-oxide (LDAO) to a final concentration of 4% and stirring for 3 h at room temperature.

Detergent-solubilized protein was purified by ion-exchange chromatography on a 250 ml POROS HQ column equilibrated with 20 m*M* Tris–HCl pH 8.5, 1% LDAO, washing first with the same buffer containing 5% elution buffer (20 m*M* Tris–HCl pH 8.5, 1% LDAO, 1 *M* NaCl) and then eluting the protein with a gradient of 5–100% elution buffer over 20 column volumes. Protein fractions with a purity of *A*_280_/*A*_830_ < 3.5 as determined by UV–Vis spectroscopy were collected and concentrated to a volume of <10 ml in a 100 kDa molecular-weight cutoff tube (Vivaspin). This was loaded in two batches onto a HiPrep 26/60 Sephacryl S-300 column (GE Healthcare) equilibrated with 20 m*M* Tris–HCl pH 8.5, 0.1% LDAO, 100 m*M* NaCl. Fractions with a purity of *A*_280_/*A*_830_ < 2.3 were collected and exchanged into final protein buffer (20 m*M* NaH_2_PO_4_/Na_2_HPO_4_ pH 6.8, 0.1% LDAO, 10 µ*M* ethylenedi­aminetetraacetic acid) by concentrating and diluting two times. The final concentration was then adjusted to 20 mg ml^−1^ before flash-freezing in liquid nitrogen and storage at −80°C.

The frozen protein was thawed, centrifuged at 16 900*g* for 15 min to remove aggregates, concentrated to 45 mg ml^−1^ as determined by UV–Vis absorption at *A*_830_ and mixed with microcrystal seeds (Dods *et al.*, 2017 ▸) in a 100:5 ratio. UQ1 or UQ2 was mixed with melted monoolein at 0.5%(*v*/*v*). The protein and lipid were then mixed in a 40:60 ratio to form the LCP. 10–15 µl of LCP was dispensed into each well of an 800 µl well plate (Hampton Research) containing 100 µl precipitant [120 m*M* 1,2,3-heptanetriol isomer T (Sigma–Aldrich), 40 m*M* zinc sulfate, 100 m*M* sodium citrate pH 6.0] and sealed with plastic film (Molecular Dimensions). Crystals appeared after two days and were fully grown after three days, at which point the LCP was collected in 500 µl gas-tight syringes (Hamilton) for storage and transport.

For the 2.4 Å resolution structure samples were transferred to the viscous injector (Shimazu *et al.*, 2019 ▸) via a 100 µl syringe and data were collected on the BL3 beamline at SACLA, Japan. Diffraction data were collected with 9.0 keV X-rays at a repetition rate of 30 Hz on a multi-port charge-coupled device (MPCCD) pixel detector (Kameshima *et al.*, 2014 ▸) with a sample-to-detector distance of 61 mm. Online sorting to separate images with diffraction from blank images was performed with *Cheetah* (Barty *et al.*, 2014 ▸) and hits were indexed with *CrystFEL* version 0.6.3 (White *et al.*, 2016 ▸) using the peak-detection method from *Cheetah*. Out of 154 600 images, 92 838 could be indexed with *DirAx* (Duisenberg, 1992 ▸), giving an indexing rate of 60.1%. Scaling and merging was performed in *CrystFEL* with orthorhombic symmetry, where scaling was performed using Monte Carlo methods in the *partialator* module. Structure factors were calculated from the intensities in *CCP*4 using *TRUNCATE* (French & Wilson, 1978 ▸) and molecular replacement was performed with *Phaser* (McCoy *et al.*, 2007 ▸) using a 1.96 Å resolution crystal structure (PDB entry 2i5n; Li *et al.*, 2006 ▸) as a model. This yielded a molecular-replacement solution with space group *P*2_1_2_1_2. The structure was refined to 2.4 Å resolution in *REFMAC*5 (Murshudov *et al.*, 2011 ▸) by 20 cycles of rigid-body refinement followed by restrained refinement and manual model building in *Coot* (Emsley *et al.*, 2010 ▸)

For the 2.25 Å resolution structure samples were transferred to the viscous injector via a 100 µl syringe and data were collected on the Alvra beamline at SwissFEL, Switzerland. Diffraction data were collected with 11.25 keV X-rays at a repetition rate of 50 Hz on a JUNGFRAU charge-integrating detector with a sample-to-detector distance of 93 mm. Online sorting to separate images with diffraction from blank images was performed using the SwissFEL data-processing pipeline (Nass *et al.*, 2021 ▸) and hits were indexed with *CrystFEL* version 0.9.0 using *XGANDALF* (Gevorkov *et al.*, 2019 ▸). Out of 260 392 images, 160 275 could be indexed, giving an indexing rate of 61.6%. Scaling and merging was performed in *CrystFEL* with orthorhombic symmetry, where scaling was performed using Monte Carlo methods in the *partialator* module. Structure factors were calculated from the intensities in *CCP*4 using *TRUNCATE* and molecular replacement was performed with *Phaser* using the previous structure as a model. The structure was refined to 2.25 Å resolution in *REFMAC*5 by 20 cycles of rigid-body refinement followed by restrained refinement and manual model building in *Coot*.

## 3. Results and discussion

### 3.1. Growth of microcrystals and microseeding

**Figure 1:**
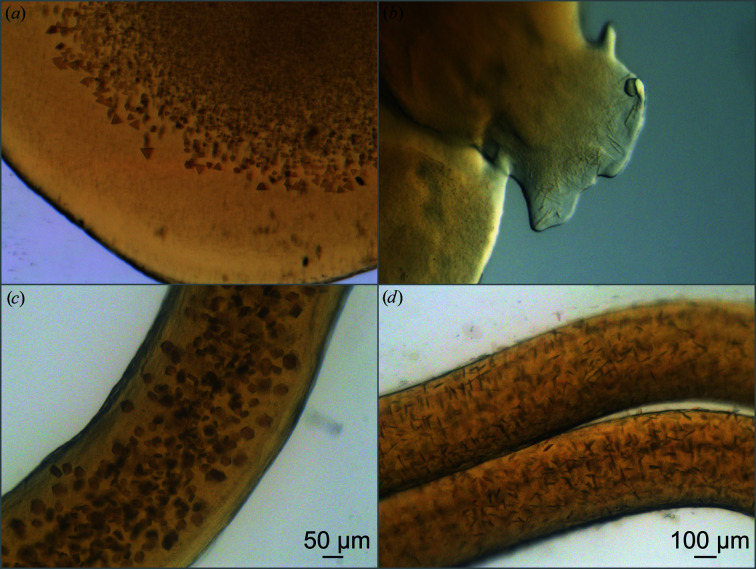
LCP microcrystallization of RC*_vir_*. (*a*) Early leads after screening. (*b*) Thin needle-like crystals which grew in syringes and diffracted to 2.4 Å resolution at SACLA but with a low hit rate. (*c*) Initial crystals grown in 800 µl glass wells. These crystals diffracted to 2.2 Å resolution at SACLA but had a long crystal axis and could not be indexed. (*d*) Seeded LCP crystals suitable for SFX and TR-SFX studies using XFEL radiation. These crystals diffracted to 2.25 Å resolution.

Initial crystal screens were set up based on previous conditions (Chiu *et al.*, 2000 ▸) and selected commercial screens developed for LCP crystallization. All conditions that gave hits (Fig. 1 ▸ *a*) included small-molecule diols or triols as additives, indicating that these amphiphiles have a similar function to heptanetriol in forming crystal contacts (Gast *et al.*, 1994 ▸). Crystallization setups in gas-tight syringes gave needle-like crystals that diffracted to 2.4 Å resolution (Fig. 1 ▸ *b*), but due to their being very thin in one direction only the largest crystals gave diffraction and the hit rate was extremely low. Screening around these conditions in crystallization plates (Andersson *et al.*, 2019 ▸) instead of syringes yielded crystals with a rhomboid-like topology with an acceptable crystal thickness in all directions (Fig. 1 ▸ *c*). These crystals diffracted to 2.2 Å resolution and the diffraction data indicated that the crystals belonged to a hexagonal space group. However, the large unit cell of the crystal prevented it from being indexed.

To improve the quality of the crystals and recover microcrystals with a smaller unit cell, we further pursued microseeding. Seeding is a common technique in vapour-diffusion crystallization that is widely used to improve crystal growth. Seeding has been less common in LCP crystallization due to difficulties in crushing LCP crystals to form a smooth distribution of crystal seeds, although innovative approaches have been applied (Kolek *et al.*, 2016 ▸). We instead developed a new protocol for seeding LCP setups to produce microcrystals for SFX based upon our earlier vapour-diffusion microcrystals (Dods *et al.*, 2017 ▸). In this approach, a seed stock was produced from crystals grown in a vapour-diffusion crystallization setup using detergent-solubilized membrane protein. These seeds were then broken into microseeds by vortexing. This microseed stock was then mixed with the protein solution before mixing with monoolein in a 40:60 ratio to form the LCP. The resulting LCP was then dispensed as 10–15 µl aliquots into an 800 µl well containing 100 µl precipitant solution (Andersson *et al.*, 2019 ▸). Although it is unconventional to use microcrystal seeds derived from vapour-diffusion crystallization to generate microcrystals in an LCP environment, the approach worked and allowed LCP microcrystals to be reliably recovered.

The density and size of the LCP microcrystals was dependent upon the ratio of monoolein to protein when forming the LCP phase, with a higher ratio of monoolein giving smaller crystals with higher density. An increase in the precipitant concentration also made the microcrystals slightly larger, but heptanetriol induces a phase transition from LCP to LSP if the concentration is too high. In contrast, the LCP microcrystal density or size did not appear to be sensitive to the concentration of the seeds. Fresh seeds, however, were essential for crystal growth, indicating that they provide nucleation sites for the crystals to grow from. Since the vapour-diffusion-grown crystals and LCP crystals have completely different packings, it seems unlikely that these seeds provided a building block for further growth, as is well known for traditional seeding. As such, we suggest that these seeds acted by providing a localized volume of highly pure and concentrated protein within the LCP from which microcrystals grew. These protocols were optimized to yield showers of plate-like crystals which were thick enough in all directions to maintain a high hit rate in SFX studies (Fig. 1 ▸ *d*), with the final data set being processed to 2.25 Å resolution.

### 3.2. Data collection and X-ray diffraction

**Figure 2:**
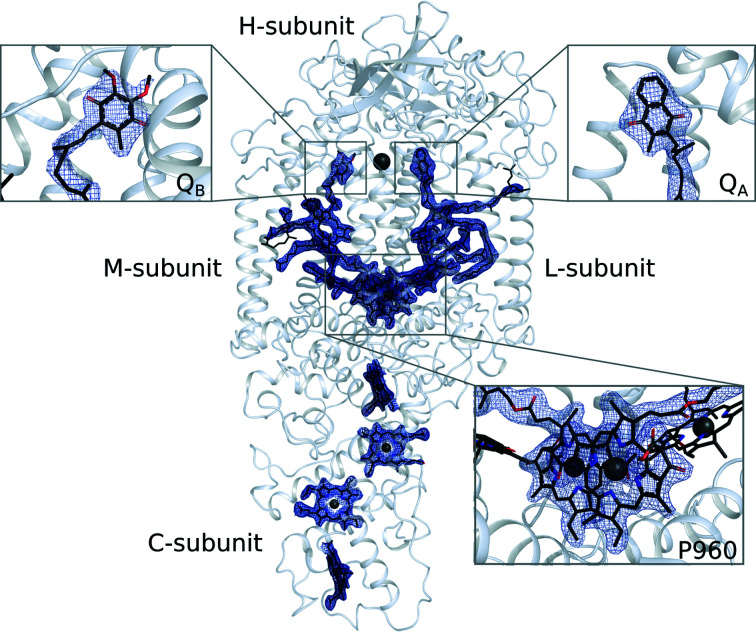
Electron density recovered for the SFX LCP structure of RC*_vir_*. Diffraction data were recorded and processed to 2.25 Å resolution. Electron density recovered for the mobile ubiquinone (Q_B_) was of similar quality to that recovered for other cofactors. 2*F*_obs_ − *F*_calc_ electron-density maps (blue) are contoured at 1σ, where σ represents the root-mean-square electron density of the map. The A-­branch leading to Q_A_ is on the right, whereas the M-branch leading to Q_B_ is on the left.

**Figure 3:**
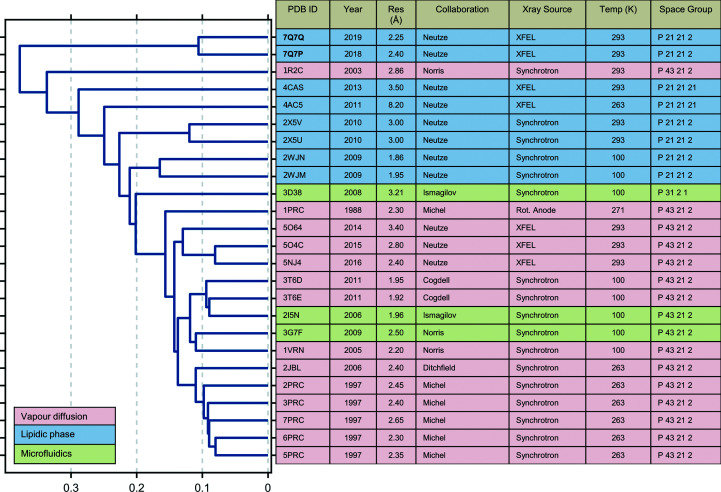
Hierarchal tree analysis of crystallographic structures of RC*_vir_* deposited in the Protein Data Bank. Crystal structures are sorted according to the mean difference of their internal distances, as described by Wickstrand *et al.* (2015 ▸). The LCP SFX structures described here (PDB entry 7q7p, data collected at SACLA; PDB entry 7q7q, data collected at SwissFEL) form their own cluster, but have a mean internal distance-matrix difference on C^α^ atoms of 0.35 Å relative to all other RC*_vir_* structures. Although the space group (*P*2_1_2_1_2) is the same as that found for earlier LCP crystal structures of RC*_vir_*, the unit cell has a significantly shorter *c* axis than that recovered in the low-temperature LCP crystal structures.

Microcrystals were transported at room temperature to SACLA, the XFEL user facility in Japan (Tono *et al.*, 2013 ▸). Data were collected from seeded crystals on the BL3 beamline of SACLA, Japan in November 2018. These microcrystals yielded diffraction to 2.4 Å resolution and approximately 93 000 images were indexed (Tables 1 ▸ and 2 ▸), which was sufficient for structural determination (Fig. 2 ▸). Interestingly, although the crystal seeds were derived from crystals grown in space group *P*4_3_2_1_2 using vapour-diffusion crystallization protocols (Dods *et al.*, 2017 ▸), the LCP crystals grew in space group *P*2_1_2_1_2, which is the same as earlier crystals grown in an LSP matrix (Wöhri *et al.*, 2009 ▸). Internal difference-matrix analysis of the position of the C^α^ atoms (Wickstrand *et al.*, 2015 ▸; Dods *et al.*, 2017 ▸) shows that the structure does not cluster closely with other earlier structures, not even LSP structures solved in *P*2_1_2_1_2 (Fig. 3 ▸), with an average difference in internal C^α^ distances of 0.35 Å relative to all other structures. On closer inspection, it is apparent that the LCP microcrystal form has a slightly different crystal packing, with crystallographic axes *a* = 84.9, *b* = 125.3, *c* = 182.7 Å. Thus, while the *a* and *c* axes are of similar length to earlier Laue diffraction structures solved in *P*2_1_2_1_2 from data collected at room temperature (*a* = 85.7, *b* = 143.5, *c* = 178.0 A; PDB entry 5x5u) and monochromatic data collection at cryogenic temperature (*a* = 84.5, *b* = 138.5, *c* = 177.8 Å; PDB entry 2wjm), the *b* axis of 125.3 Å is 20 Å and 13 Å shorter than in these two other cases, respectively. A second set of data was also collected at the SwissFEL in Switzerland in October 2019. By collecting significantly more data, 160 000 images could be indexed and the resolution was improved to 2.25 Å. The unit cell (*a* = 84.7, *b* = 125.1, *c* = 182.4 Å) is very similar to the SACLA RC*_vir_* structure and clusters with it in the internal distance-matrix analysis (Wickstrand *et al.*, 2015 ▸). Thus, differences in crystal packing have a larger effect on the refined X-ray structure than the chosen data-collection strategy. Both LSP and LCP *P*2_1_2_1_2 crystal forms have similar crystal packing, with both growing as stacked 2D crystals, but the shorter *b* axis of the LCP form appears to give a slightly tighter packing.

### 3.3. Occupancy of Q_B_ and the position of ubiquinone

**Figure 4:**
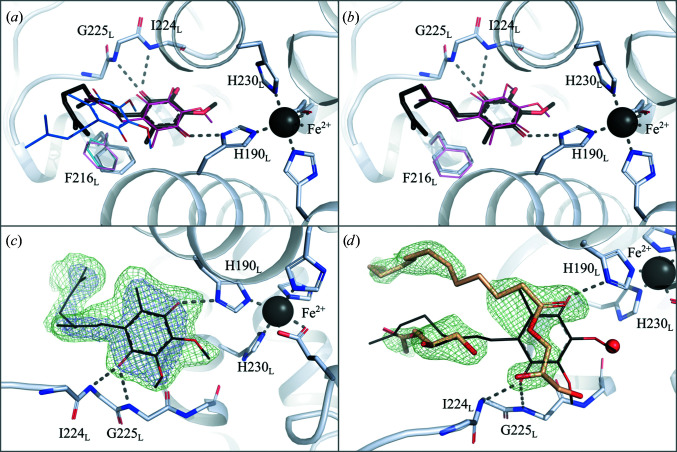
Structure of the mobile ubiquinone Q_B_ binding site. (*a*) Superposition of ubiquinone observed here (black) with earlier proximal (red) and distal (blue) Q_B_ binding sites in RC_Sph_ (PDB entries 1aig and 1aij). (*b*) Superposition of ubiquinone observed here (black) with an earlier proximal (red) Q_B_ binding site in RC*_vir_* (PDB entry 2i5n). (*c*) Electron density for the LCP SFX structure. (*d*) Electron density showing the presence of monoolein in an earlier LSP crystal form (PDB entry 2wjn). *F*_obs_ − *F*_calc_ omit electron-density maps (green) are contoured at 3σ. 2*F*_obs_ − *F*_calc_ electron-density maps (blue) are contoured at 1σ.

One potential advantage of crystallizing in LCP is the possibility of adding ubiquinone to the crystallization setup, as it easily mixes into monoolein. In contrast with previous LSP structures, in which the native ubiquinone competed unsuccessfully with monoolein (Selikhanov *et al.*, 2020 ▸; Wöhri *et al.*, 2009 ▸; Fig. 4 ▸ *d*), UQ1 or UQ2 were doped into the monoolein in our crystallization condition and yielded high occupancy in the Q_B_ pocket (Fig. 4 ▸ *c*). Indeed, the quality of the electron density in this region was comparable with that of other cofactors within the protein (Fig. 2 ▸). When Q_B_ is modelled with 100% occupancy its average *B* factor is 60.9 Å^2^, which compares with an average *B* factor of 39.0 Å^2^ for Q_A._ The additional mobility of Q_B_ may explain this difference in apparent *B* factors, although it is equally possible that the occupancy is somewhat lower than 100% but nevertheless well above 50%.

Earlier crystallographic studies indicated that the Q_B_ ligand can populate two different positions in its binding pocket: a proximal position closer to the nonhaem iron as well as a distal position that is further removed (Fig. 4 ▸ *a*), where only the proximal position allows electrons to be transferred to the uniquinone (Stowell *et al.*, 1997 ▸). In the SFX LCP structure, UQ occupies the proximal position next to the iron, where it can form hydrogen bonds to the N atoms of His190_L_, Ile224_L_ and Gly225_L_ (Fig. 4 ▸ *a*). When compared with the structure of the reaction centre from *Rhodobacter sphaeroides* (RC_Sph_; Stowell *et al.*, 1997 ▸) the head group of Phe216_L_ is slightly displaced (Fig. 4 ▸ *a*) to facilitate ubiquinol binding to the proximal position. As in an earlier structure showing native UQ9 in the RC*_vir_* binding pocket (Li *et al.*, 2006 ▸; Fig. 4 ▸ *b*), the UQ tail is very flexible and only the first isoprene unit can be modelled with certainty.

It was previously thought that the distal ubiquinone-binding position was the predominant form in RC*_vir_* as it provided a better fit to the electron density observed in earlier structures (Lancaster & Michel, 1997 ▸). However, the idea that Q_B_ moves between the distal and proximal positions in response to light as a gating mechanism for electron transfer (Stowell *et al.*, 1997 ▸; Fritzsch *et al.*, 2002 ▸) has been questioned from FTIR spectroscopy studies (Breton, 2004 ▸). Moreover, freeze-trap studies of illuminated and dark RC_Sph_ crystals show that the pH of the crystal conditions influences the distribution between the distal and proximal positions (Koepke *et al.*, 2007 ▸) and a comparison of dark and illuminated crystals shows ubiquinone predominantly binding to the proximal position if the pocket is occupied at all (Baxter *et al.*, 2005 ▸).

### 3.4. A second ubiquinone-binding site

**Figure 5:**
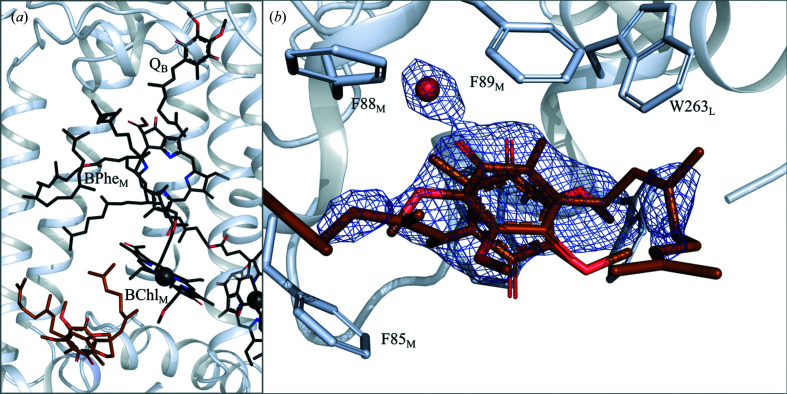
Structure of the surface-bound ubiquinone molecule. (*a*) The location of the surface-bound ubiquinone molecule is indicated in orange. (*b*) 2*F*_obs_ − *F*_calc_ electron-density map showing electron density for this ubiquinone. Two possible conformations are shown since the electron-density map does not yield a unique orientation for the surface-bound ubiquinone molecule. This map is contoured at 1σ.

In the SFX LCP RC*_vir_* structure, a second ubiquinone-binding site could be observed on the same side of the protein as the Q_B_ pocket but closer to the C subunit. Specifically, the ubiquinone binds within a hydrophobic pocket sandwiched between Phe89_M_ and Trp263_L_ (Fig. 5 ▸). Although the crystallographic occupancy of this quinone is lower than for the Q_B_ pocket (a *B* factor of 91 Å^2^ is recovered when modelled with 100% occupancy and this is more than twice that of the Q_A_ site) this feature has been observed in several other crystal structures of RC*_vir_* [PDB entries 2i5n (Li *et al.*, 2006 ▸), 3g7f (Ponomarenko *et al.*, 2009 ▸), 3t6d and 3t6e (Roszak *et al.*, 2012 ▸), 3d38 (Li *et al.*, 2008 ▸) and 7pil (Qian *et al.*, 2021 ▸)]. Moreover, both Trp263_L_ and Phe89_M_ are conserved residues between species (Roszak *et al.*, 2012 ▸), but the functional role of this binding site is unclear. A recent cryo-EM structure of RC*_vir_* in complex with light-harvesting complex 1 did not show this ubiquinone binding. Instead, a ubiquinone is modelled close to the proposed entry through the LH ring in the Q_A_ site of the RC (Qian *et al.*, 2018 ▸). It therefore seems reasonable to believe that this second ubiquinone site is one of many in a dynamic environment as the ubiquinone traverses the membrane, but is nevertheless stable enough to let the ligand remain bound to the protein during crystallization.

### 3.5. High flexibility of bound lipids

**Figure 6:**
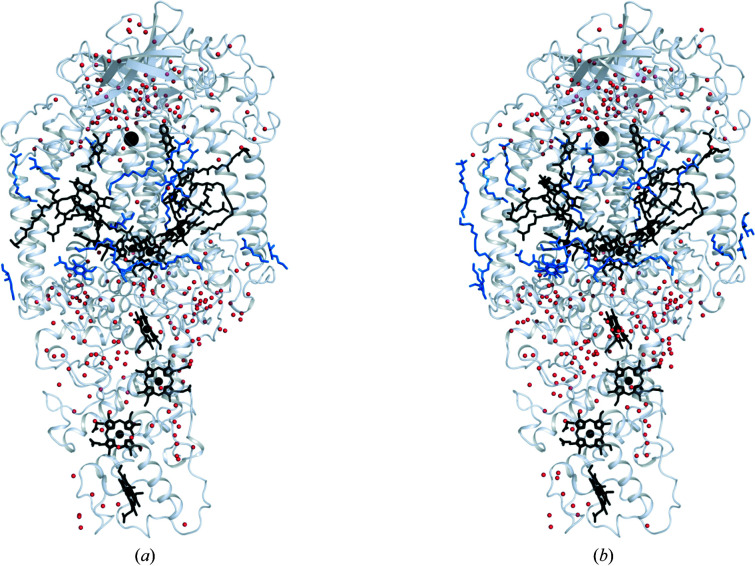
A comparison of the waters (red) and lipids (blue) distributed within the two presented structures, with (*a*) showing the 2.4 Å resolution structure and (*b*) showing the 2.25 Å resolution structure. There are more lipids visible in (*b*), especially on the left side of the image corresponding to one of the contacts between the proteins within the crystal structure.

Within the transmembrane region, the tails of the two bound ubiquinone molecules reach into a highly flexible region where several other cofactors also lack electron density. This includes the tails of the M-branch cofactors BChl_M_ and BPhe_M_ as well as the tail of the 1,2-dihydroneurosporene carotenoid. Moreover, the diacyl glycerol molecule which is fused to the C-terminus of the C subunit also has poor electron density for its dual acyl tails, and only one of them is modelled in the 2.25 Å resolution structure as the remaining atoms are undefined. This is consistent with previous SFX structures, for which the extent of radiation damage was low (Dods *et al.*, 2017 ▸; Johansson *et al.*, 2013 ▸). Fewer detergent molecules are also modelled than are usually identified in comparable low-temperature single-crystal X-ray structures of RC*_vir_*. While some of these differences may be due to differences in crystal packing, it is also possible that these differences arise from the additional flexibility of the membrane region during data collection at room temperature. A groove near the Q_A_ pocket also contains two LDAO molecules in our vapour-diffusion microcrystals (Dods *et al.*, 2017 ▸) and a phospholipid in the LSP crystal form (Wöhri *et al.*, 2009 ▸). In the XFEL structures reported here, electron density in these regions is modelled either by an LDAO or a monoolein molecule, but the occupancy is lower than for the vapour-diffusion crystal structure and one of the LDAOs in that structure is not visible at all. As in our vapour-diffusion microcrystals, the number of visible lipids increases with the resolution, and the 2.25 Å resolution structure is modelled with five extra detergent molecules and 23 extra water molecules when compared with the 2.4 Å resolution structure (Fig. 6 ▸). In the 2.25 Å resolution structure Q_A_ could be modelled with an additional carbon in the tail of the molecule and the lipid crystal contacts between the two M subunits are more defined.

## 4. Conclusion

Over the last decade, serial crystallo­graphic studies using XFEL radiation have progressed from consuming millilitres of microcrystal slurries to the collection of full data sets with the consumption of only microlitres of microcrystal slurries. To a large extent, this development is due to the possibility of using LCP (Weierstall *et al.*, 2014 ▸) or grease (Sugahara *et al.*, 2015 ▸) as a carrier medium. There have also been improvements in the screening of crystals through automated pipetting robots, including dedicated systems for LCP setups. Moreover, serial crystallography is also being developed on microfocus beamlines at synchrotron user facilities (Nogly *et al.*, 2015 ▸), which allow the collection of data sets both for structural determination and time-resolved diffraction studies (Mehrabi *et al.*, 2019 ▸). Crystallization of RC_*vir*_ has shifted greatly since the first structures in the 1980s (Deisenhofer *et al.*, 1984 ▸, 1985 ▸), which used very large crystals measuring millimetres in size, to now using microcrystals at XFEL sources. By extending our method of microseeding (Dods *et al.*, 2017 ▸) to LCP microcrystallization, we have developed a crystallization protocol utilizing an unconventional seeding approach that yielded well diffracting LCP microcrystals with excellent Q_B_ occupancy. These new conditions provide a starting point for both TR-SFX studies in the sub-picosecond time domain (Nogly *et al.*, 2018 ▸; Barends *et al.*, 2015 ▸; Pande *et al.*, 2016 ▸) as well as slower studies of the two electron-transfer reactions in RC*_vir_* using XFEL or synchrotron radiation.

## Supplementary Material

PDB reference: *Blastochloris viridis* photosynthetic reaction centre, 7q7p

PDB reference: 7q7q

## Figures and Tables

**Table 1:** Data collection and processing Values in parentheses are for the outer shell.

	SACLA	SwissFEL
Wavelength (Å)	1.38	1.10
Temperature (K)	293	293
Crystal-to-detector distance (mm)	61	93
Space group	*P*2_1_2_1_2	*P*2_1_2_1_2
*a*, *b*, *c* (Å)	84.9, 125.3, 182.7	84.7, 125.1, 182.4
α, β, γ (°)	90, 90, 90	90, 90, 90
Resolution range (Å)	73.92–2.40 (2.42–2.40)	23.7–2.25 (2.27–2.25)
Total No. of reflections	54264823	110014210
No. of unique reflections	76977	92545
Completeness (%)	100.0 (100.0)	100.0 (100.0)
Multiplicity	705 (286)	1189 (838)
〈*I*/σ(*I*)〉	7.9 (1.1)	9.6 (1.2)
*R*_split_ †	10.6 (106)	7.9 (88)
Overall *B* factor from Wilson plot (Å^2^)	46.7	49.0

† *R*_split_ = 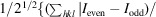 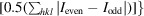 .

**Table 2:** Structure solution and refinement Values in parentheses are for the outer shell.

	SwissFEL (PDB entry 7q7q)	SACLA (PDB entry 7q7p)
Resolution range (Å)	23.7–2.25 (2.31–2.25)	73.0–2.40 (2.46–2.40)
Completeness (%)	99.86	99.95
No. of reflections, working set	87913	73102
No. of reflections, test set	4602	3808
Final *R*_cryst_	0.168	0.165
Final *R*_free_	0.203	0.204
No. of non-H atoms		
Protein	9297	9286
Ligand	396	381
Water	267	244
R.m.s.d.		
Bond lengths (Å)	0.008	0.014
Angles (°)	2.132	2.373
Average *B* factor (Å^2^)	51	49
Ramachandran plot		
Most favoured (%)	94.8	94.14
Allowed (%)	4.4	5.17
